# TCGASpliceSeq a compendium of alternative mRNA splicing in cancer

**DOI:** 10.1093/nar/gkv1288

**Published:** 2015-11-23

**Authors:** Michael Ryan, Wing Chung Wong, Robert Brown, Rehan Akbani, Xiaoping Su, Bradley Broom, James Melott, John Weinstein

**Affiliations:** 1 Department of Bioinformatics and Computational Biology, The University of Texas MD Anderson Cancer Center, Houston, TX 77030, USA; 2 In Silico Solutions, Falls Church, VA 22043, USA; 3 Department of Systems Biology, The University of Texas MD Anderson Cancer Center, Houston, TX 77030, USA

## Abstract

TCGA's RNASeq data represent one of the largest collections of cancer transcriptomes ever assembled. RNASeq technology, combined with computational tools like our SpliceSeq package, provides a comprehensive, detailed view of alternative mRNA splicing. Aberrant splicing patterns in cancers have been implicated in such processes as carcinogenesis, de-differentiation and metastasis. TCGA SpliceSeq (http://bioinformatics.mdanderson.org/TCGASpliceSeq) is a web-based resource that provides a quick, user-friendly, highly visual interface for exploring the alternative splicing patterns of TCGA tumors. Percent Spliced In (PSI) values for splice events on samples from 33 different tumor types, including available adjacent normal samples, have been loaded into TCGA SpliceSeq. Investigators can interrogate genes of interest, search for the genes that show the strongest variation between or among selected tumor types, or explore splicing pattern changes between tumor and adjacent normal samples. The interface presents intuitive graphical representations of splicing patterns, read counts and various statistical summaries, including percent spliced in. Splicing data can also be downloaded for inclusion in integrative analyses. TCGA SpliceSeq is freely available for academic, government or commercial use.

## INTRODUCTION

Alternative mRNA splicing is a regulated process that occurs in nearly all multi-exon human genes (1). The splicing mechanism enables a single gene to produce multiple protein products, and changes in splicing patterns are closely associated with development and differentiation. Of particular interest to cancer researchers are the alternate splicing changes in tumor cells that may represent a return to developmental splicing patterns of metabolism, angiogenesis, mobility and proliferation (2,3). The Cancer Genome Atlas (TCGA) project has generated a very large, public collection of multiple types of ‘omic’ data on samples from >10,000 cancer patients, across 33 different tumor types. The TCGA data provide a rich source for the investigation of alternative splicing patterns in cancer.

In practice, however, integrating TCGA splicing data into broader analytical investigations is a prohibitively complex, compute-intensive process for most cancer genomics researchers. Exon, splice and transcript isoform expression levels are available from the TCGA Data Portal but do not come with: a unified gene model to support comparative analysis, splice event measurements, tumor specific summarizations, splice pattern graphical displays, protein impact information or simple query/download features for integrative analysis. To address these needs, we used SpliceSeq (4) to analyze thousands of TCGA RNASeq samples, computing and storing the consequent statistics in a TCGA splicing database. The splicing data are available through our TCGA SpliceSeq website. The site presents splicing patterns and related statistics in an intuitive, interactive, graphical form with dynamic features for exploration of cross-tumor-type or tumor-normal splice variations. TCGA SpliceSeq's data query/download capability enables researchers to retrieve per-sample, per-splice event measures of alternative splicing that can then be included in any type of integrative analysis. It also provides search functions that identify splice event variation across selected tumor types. Hence, it can be used to focus on particular genes, or, in knowledge discovery mode, to identify the most striking splice events.

TCGA SpliceSeq is freely available for academic, government or commercial use at http://bioinformatics.mdanderson.org/TCGASpliceSeq/.

## DATABASE CONSTRUCTION

**Figure 1.**
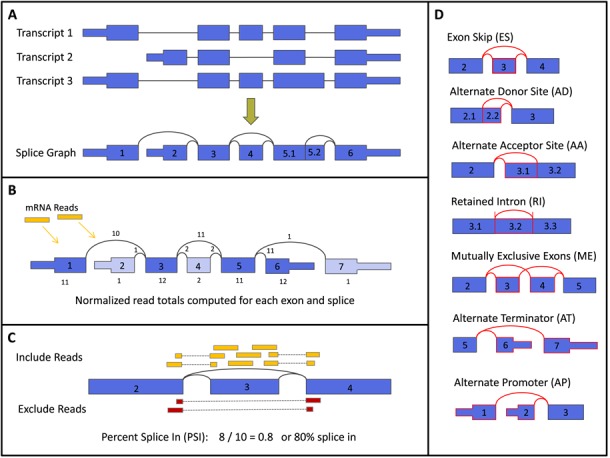
SpliceSeq analysis of mRNA data. (**A**) Ensemble coding transcripts for each gene are assembled into a unified splice graph. (**B**) Reads are aligned to the splice graphs and totals for each exon and splice are calculated. Read counts are normalized to the length of each exon and number of aligned reads in the sample. (**C**) Percent Spliced In values are computed for each possible splice event in each gene. PSI is the ratio of reads indicating the presence of a transcript element versus the total reads covering the event. In this example, the 8 yellow reads (exon 3 body reads, exon 2–3 junction reads and exon 3–4 junction reads) indicate that exon 3 is present. The red junction 2–4 reads indicate that exon 3 was spliced out. The PSI is therefore 8/10 or 0.8 indicating that 80% of the transcripts in the sample include exon 3. (**D**) SpliceSeq evaluates all of the listed types of splice events.

We used our SpliceSeq tool to analyze the mRNA splicing patterns of TCGA samples. SpliceSeq starts with a reference model for each gene constructed from all of the gene's protein-coding transcripts in the Ensembl gene database (5). The transcript isoforms of each gene are assembled into a unified splice graph such that mRNA reads will align to a single unique location in the graph (Figure 1). TCGA sample reads are aligned to the splice graphs, and summary statistics are generated for each exon and splice junction. Read totals are normalized by exon length and the number of aligned reads in the sample (reads per 1000 bases of exon per million aligned reads). A second pass alignment is performed on unaligned reads looking for novel splices not present in the original Ensembl models. Novel exons are not identified by this algorithm.

The Percent Spliced In (PSI) value is a common, intuitive ratio for quantifying splicing events (1). The number of reads indicating that a transcript element is present is divided by the total number of reads covering the splicing event. In the example of an exon skip event in Figure 1C, a PSI value of 0.8 indicates that approximately 80% of the transcripts in the sample include exon 3 and 20% do not. PSI values were calculated for each potential splice event on all genes expressed in the TCGA samples. There are seven types of splice events: exon skip, alternate 5′ donor, alternate 3′ acceptor, retained intron, mutually exclusive exons, alternate first exon and alternate last exon.

The results of SpliceSeq analysis of TCGA samples are stored in the TCGA SpliceSeq relational database. To date, we have fully loaded 15 tumor types, the 12 included in the first TCGA pan-can analysis and 3 additional tumor types. The fully loaded data contain PSI values for 80,000+ splice events on 19,036 genes for 6238 tumor samples and 496 adjacent normal samples. Aligning and loading the data is a computationally intensive task. For TCGA tissues that have not yet been fully loaded into the database, we analyzed 15 randomly selected tumor and 15 randomly selected adjacent normal samples (when available) to provide an initial overview of splicing in these tumor types. As additional full data sets are loaded, they will replace the sub-sampled data. Full data sets are indicated by a ‘***’ next to the tumor type on the PSI Data Download page.

PSI values are not provided for a sample unless at least 8 reads cover the event (include + exclude reads ≥ 8). Novel splices are those that were not included in the reference Ensembl gene models but were detected in our second pass alignment. These can be spurious or very weakly expressed so if a splice event includes a novel splice, PSI values for the event will only be reported if the novel splice is expressed in ≥ 1% of transcripts of 10 or more samples.

## DATABASE USAGE

The TCGA SpliceSeq database can be queried and explored via our graphical web application at http://bioinformatics.mdanderson.org/TCGASpliceSeq.

### Investigate a gene of interest

**Figure 2.**
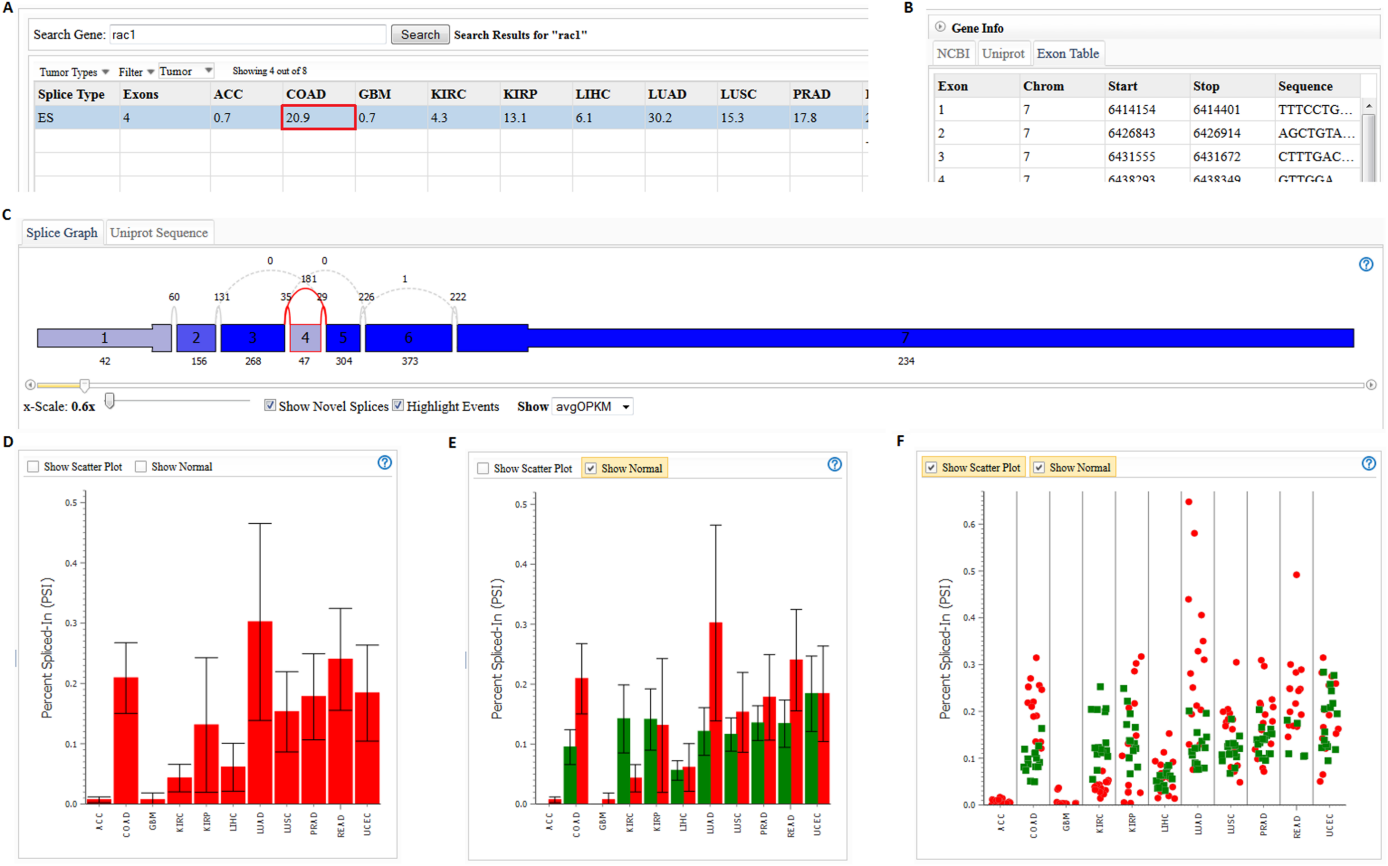
TCGA SpliceSeq gene symbol search results. (**A**) Results table in the TCGA SpliceSeq gene symbol search. Each row contains data for a splice event in the gene. The table columns show the average PSI values for specific tumor types. Selecting a cell for a splice event/tumor type (e.g. exon 4 skip event in COAD) causes all other display panels to present data for the selected event or tissue. (**B**) The gene information tab provides a general description of the selected gene and the hg19 coordinates and sequence for each exon. The exon information can be used to identify exons mentioned in literature with different naming schemes. (**C**) A splice graph of the gene's exons is shaded based on expression level and shows the selected splice event outlined in red. **(D)**The PSI plot for the exon 4 skip event in *RAC1* shows the expected inclusion of exon 4 in COAD but also strong use of exon 4 in LUAD and several other tissues. (**E**) Selecting ‘Show Normal’ in the plot panel shows PSI values for the same splice event in adjacent normal tissue. Normal values show that the exon is expressed in normal tissues but that COAD and LUAD tumor samples have higher exon 4 expression than adjacent normal. (**F**) Selecting ‘Show Scatter Plot’ in the plot panel displays individual sample PSI values, providing insight into the range/consistency of PSI values for a group of samples. LUAD samples demonstrate a much wider range of exon 4 inclusion than do COAD samples.

The splicing patterns of a specific gene in a variety of tumor types can be investigated by entering the gene symbol on the TCGA SpliceSeq home page or on the Single Gene query tab. For example, the *RAC1* gene has an alternate exon (exon 4 in our *RAC1* splice graph) that is strongly conserved but not included in the canonical isoform, suggesting a potential role of the exon in early development. Inclusion of this exon was found to be increased in colorectal cancer and to play a role in metastasis (2). Querying *RAC1* in TCGA SpliceSeq returns a table (Figure 2A) of PSI values for each splice event for each TCGA tumor type. In this case, we are interested in the first row of the table related to the inclusion/skip of exon 4. A table containing the hg19 chromosomal location and sequence of exons (Figure 2B) is provided to help clarify splice event details, as the SpliceSeq exon numbering may differ from exon naming from other sources.

Clicking on a PSI value in the table causes the splice graph (Figure 2C) to highlight the location of the splice event in red and display the average expression pattern for the selected tissue type. Each box in the splice graph represents an exon, and each arc represents a splice. Narrow exon sections are untranslated regions and thicker exon sections are protein coding. Exons that are dark blue are expressed at the highest level relative to the overall gene expression level. The numerical values adjacent to each exon and splice provide raw or normalized read counts.

A plot of the PSI values across tumor types (Figure 2D) is also displayed based on the splice event selected in the results table. This plot provides an informative overview of the splice event expression pattern across different tumor types. In the case of the *RAC1* exon 4 skip event in the figure, the TCGA data show that the exon is indeed expressed in colon tumors (COAD) as reported in the literature. We can also see that the exon is not expressed in adrenocortical carcinoma (ACC) or glioblastoma multiforme (GBM) but is expressed in a variety of other tumor types, including rectal adenocarcinoma (READ)—as expected because it is very similar to COAD—and at even higher levels in lung adenocarcinoma (LUAD). This quick exploration suggests that the metastatic activity of *RAC1* exon 4 inclusion in COAD may be worth exploring in LUAD and other tumors.

The ‘Show Normal’ check box on the PSI plot can be used to show the PSI values of adjacent normal samples in green (Figure 2E). The plot of normal PSI values is useful in the *RAC1* example where we see that there is expression of exon 4 in normal tissue but that the COAD, LUAD and READ tumor samples show robust increases in exon 4 inclusion. Finally, the ‘Show Scatter Plot’ check box can be used to get a more detailed view of the range of PSI values (Figure 2F). In the *RAC1* example, the COAD data show a fairly consistent shift between the tumor and normal samples, whereas the LUAD tumor samples show a very wide range of exon 4 inclusion—with a few in the same range as normal samples plus some with dramatically higher PSI values.

### Splice variation knowledge discovery

TCGA SpliceSeq allows for knowledge discovery via genome-wide PSI splice event searches to locate significant splice variation among tumor types or between tumor and normal tissue. Strong differences in splicing pattern may provide biological insights that shed light on tumor development and progression. Alternatively, splice variation may represent potential tumor-specific biological markers with clinical application as diagnostic markers or therapeutic targets.

**Figure 3.**
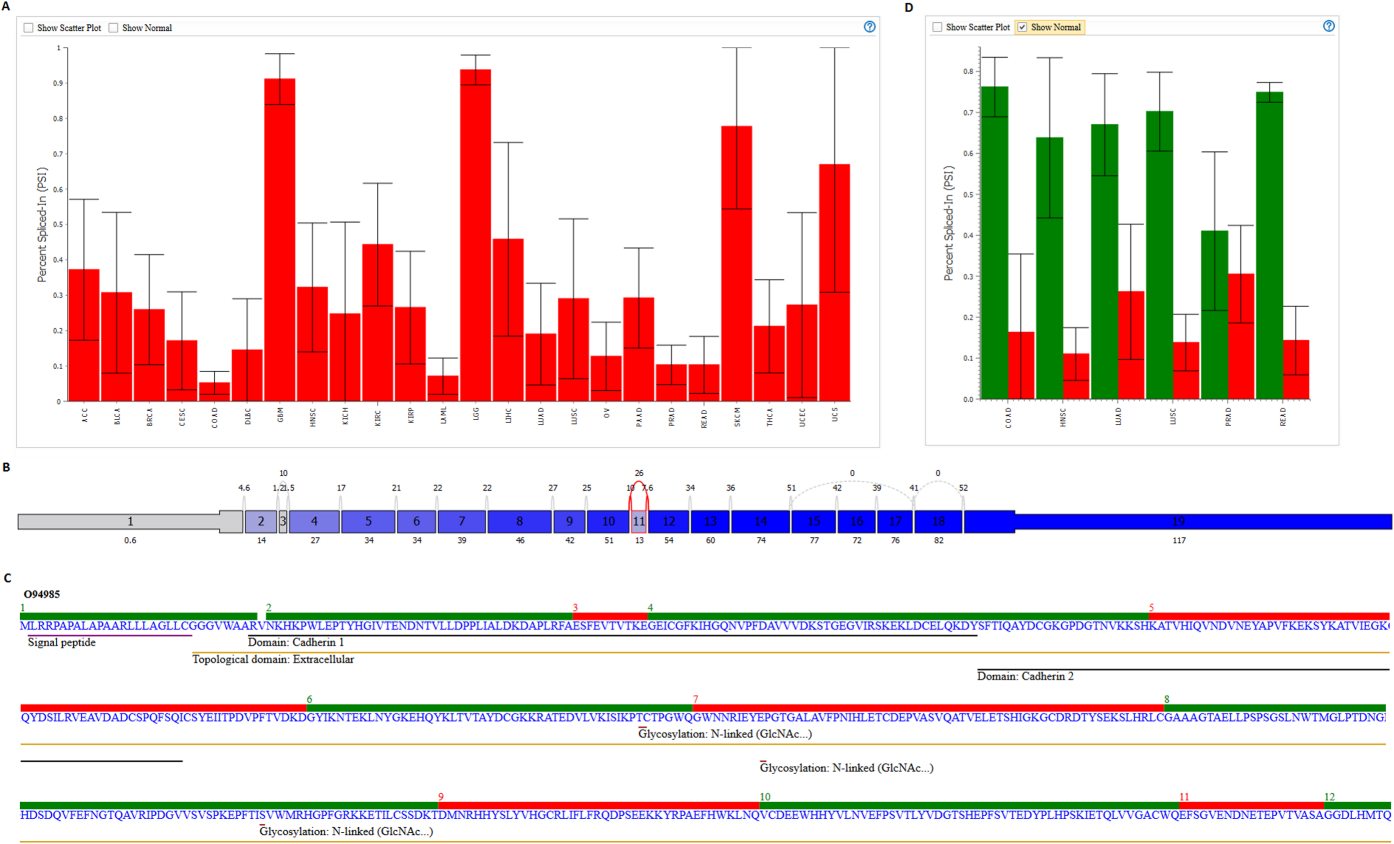
TCGA SpliceSeq Top Hits results. (**A**) PSI values for an exon 11 skip event in the *CLSTN1* gene. This gene is a top hit in the tumor difference query for all tissues because the PSI values for exon 11 inclusion vary dramatically across different cancer types. (**B**) Splice graph of the *CLSTN1* exon 11 skip event (highlighted in red) showing average expression in BLCA tumor samples. (**C**) Portion of the UniProt tab for *CLSTN1*. Red/Green sections with numbers indicate the exon that codes for each section of the protein. Tracks below the amino acid sequence show functional and structural annotations. The exon 11 skip event falls in the Extracellular region of the protein so it may affect protein–protein interactions. (**D**) PSI values of exon 11 skip event in the extracellular matrix gene *FBLN2*. The event was high in the tumor-normal results for the subset of tumor types displayed because the PSI shifts strikingly and consistently between tumor and adjacent normal values.

The Top Events page is used for knowledge discovery searches. The user selects either Tissue Difference for variation across tumor types or Tumor-Normal for splice events that show a shift in splicing patterns between tumor and adjacent normal samples. Clicking on a cell in the table for a gene and splice event causes the plot window to display PSI values for the event across all tumor types. The list returned is sorted so that the events with the highest level of variance across tumors (Figure 3A) or between tumor and normal (Figure 3D) are first in the list. If the Tumor Types dropdown menu is used to select particular cancer types of interest, the list is rebuilt to find the events with the most significant variation for the selected tumor types.

When a PSI value for a particular tissue is selected in the results table, a splice graph for the gene is displayed, with red highlighting to indicate the position of the selected splice event; the graph then shows the average exon/splice expression pattern for the selected tissue type (Figure 3B). Selecting the UniProt Sequence tab in the Splice Graph window displays the protein sequence for the selected gene with alternating green/red region markers above the sequence to indicate the exon that codes for each section of the protein (Figure 3C). Below the amino acid sequence are functional and structural annotations that may provide insight on the impact of alternative splicing.

### Download PSI data for integrative analysis

TCGA SpliceSeq allows users to investigate relationships between mRNA splicing patterns and other genomic alterations. Researchers are often interested in the relationship between splicing changes and expression levels of other genes that could regulate splicing or that participate in biological pathways impacted by splicing changes. Investigators may also be interested in exploring the associations between splicing changes and other genomic variation including mutations, DNA copy number, DNA methylation, miRNA expression and protein expression, among others. The TCGA studies produce all of these types of data. TCGA SpliceSeq supports integrative analysis by enabling users to download sample-specific PSI values that can then be used with other publicly available TCGA data for custom analyses. The interactive visualizations in TCGA SpliceSeq plot a random sampling of data from each tumor type but the download feature provides PSI values for all TCGA samples.

For example, an investigator may be interested in the relationship between the splicing regulator hnRNP A1 and the mutually exclusive exon splicing in pyruvate kinase M (*PKM*) associated with the shift to aerobic glycolysis in tumors (Warburg effect) in lung adenocarcinoma (2). To address that custom analysis, the TCGA SpliceSeq Download Data page can be used to request a download of splicing events in *PKM* for the LUAD tissue type. This will provide data for all splicing events on the gene or else the download can be limited further to a specific event type (in this case, mutually exclusive exons). The result is a tab-delimited file download of the individual cancer and related adjacent normal samples, with the Percent Sliced In (PSI) values for each sample/splice event. A custom bioinformatics analysis can then be performed using sample specific expression and splicing data to address the question of association of hnRNP A1 expression and *PKM* mutually exclusive splicing. Also available for download on the FAQ page is reference file that contains the hg19 chromosomal coordinates of each exon in the SpliceSeq gene models, enabling cross referencing of splice events with external gene models or exon naming schemes.

## LIMITATIONS AND FUTURE PLANS

Molecular analysis of solid tumors is often challenging because each tumor sample can be polyclonal and because different samples contain varying levels of stromal tissue. In addition, the TCGA adjacent normal samples are not always well matched to the tumor progenitor cells. For example, bladder urothelial carcinoma originates in epithelial cells, whereas an adjacent normal sample often contains large amounts of muscle tissue. Alternative splicing analysis, in particular, is sensitive to tissue type differences, as shifts in splicing patterns are often a key biological mechanism for differentiation. Care must be taken when interpreting TCGA splicing data to understand that tumor purity and tissue mismatched ‘normals’ may confound analysis.

The TCGA SpliceSeq database includes only alternative splicing events for protein coding genes. It does not at this time include non-coding RNAs which are known to exhibit alternate splice forms that play a role in cancer progression.

There are several additional features that we plan to add to TCGA SpliceSeq in the near future. We will add support for dynamic differential analysis of user-specified sample groups. Such a capability could be used, for example, to identify the statistically significant splicing pattern differences between basal versus luminal breast cancer or high versus low *hnRNPA1* expressors. We also plan to expand the data download capabilities to allow retrieval of additional statistics such as exon/splice raw and normalized read counts. This capability would enable bioinformatics researchers to develop their own methods for identification of splicing pattern differences. Finally, we will provide a per gene/per tumor web services interface to get sample level PSI values. The web service will provide an easy method for integrating TCGA SpliceSeq data into other bioinformatics pipelines or websites.
